# Crystal Structure of StnA for the Biosynthesis of Antitumor Drug Streptonigrin Reveals a Unique Substrate Binding Mode

**DOI:** 10.1038/srep40254

**Published:** 2017-01-11

**Authors:** Tianle Qian, Jing Wo, Yan Zhang, Quanwei Song, Guoqiang Feng, Ray Luo, Shuangjin Lin, Geng Wu, Hai-Feng Chen

**Affiliations:** 1 State Key Laboratory of Microbial metabolism, School of Life Sciences and Biotechnology, Shanghai Jiao Tong University, 800 Dongchuan Road, Shanghai, 200240, China; 2 Shanghai Institute of Immunology, Shanghai Jiao Tong University School of Medicine, 280 South Chongqing Road, Shanghai, 200025, China; 3 Key laboratory of Pesticide and Chemical Biology of Ministry of Education, College of Chemistry, Central China Normal University, 152 Luoyu Road, Wuhan 430079, China; 4 Departments of Molecular Biology and Biochemistry, Chemical Engineering and Materials Science, and Biomedical Engineering, University of California, Irvine, California 92697-3900, USA; 5 Shanghai Center for Bioinformation Technology, 1278 Keyuan Road, Shanghai, 200235, China

## Abstract

Streptonigrin methylesterase A (StnA) is one of the tailoring enzymes that modify the aminoquinone skeleton in the biosynthesis pathway of *Streptomyces* species. Although StnA has no significant sequence homology with the reported α/β-fold hydrolases, it shows typical hydrolytic activity *in vivo* and *in vitro*. In order to reveal its functional characteristics, the crystal structures of the selenomethionine substituted StnA (SeMet-StnA) and the complex (S185A mutant) with its substrate were resolved to the resolution of 2.71 Å and 2.90 Å, respectively. The overall structure of StnA can be described as an α-helix cap domain on top of a common α/β hydrolase domain. The substrate methyl ester of 10′-demethoxystreptonigrin binds in a hydrophobic pocket that mainly consists of cap domain residues and is close to the catalytic triad Ser185-His349-Asp308. The transition state is stabilized by an oxyanion hole formed by the backbone amides of Ala102 and Leu186. The substrate binding appears to be dominated by interactions with several specific hydrophobic contacts and hydrogen bonds in the cap domain. The molecular dynamics simulation and site-directed mutagenesis confirmed the important roles of the key interacting residues in the cap domain. Structural alignment and phylogenetic tree analysis indicate that StnA represents a new subfamily of lipolytic enzymes with the specific binding pocket located at the cap domain instead of the interface between the two domains.

Streptonigrin (STN, **1**, Fig. 1) is a highly functionalized aminoquinone antitumor antibiotic produced by *Streptomyces flocculus*^1^. STN was first reported in 1959^1^, and its molecular structure was initially established via spectroscopy and chemical degradation in 1963^2^. Its accurate structure was later confirmed by X-ray diffraction and ^13^C NMR analysis in 1974–75^3^,^4^. STN is composed of four aromatic rings: the aminoquinolinone rings (A and B) and pyridine (C) ring are nearly coplanar, and the multisubstituted phenyl ring (ring D) is perpendicular to rings A, B and C^4^ (Fig. 1). STN has a wide range of anti-tumor activities, effective on breast cancer, lung cancer, head and neck cancer, lymphomas, and melanomas^5^,^6^,^7^,^8^. In addition, it also shows *in vivo* and *in vitro* antiviral properties and potent, broad spectrum antibacterial activities^7^,^8^.

STN’s unique structure and potential in cancer therapy have attracted considerable attention from the cancer research community. Due to the low production in chemical synthesis^9^, Lin and co-workers identified the biosynthetic pathways of STN in 2013^10^. The biosynthetic gene cluster of STN was found to consist of 48 genes via a series of gene inactivation experiments. The targeted gene disruption experiments have shown that inactivation of *stnA* gene completely abolished production of **1** but accumulated three new compounds (**2**, **3**, and **4**). Therefore, the left boundary of this gene cluster was determined as *stnA*^10^. Although StnA shows no significant sequence homology with reported α/β-fold hydrolases, it is proposed to be an α/β-fold hydrolase that hydrolyzes the methyl ester of **4** towards the production of **5** 10.

The α/β-fold hydrolases family of enzymes is one of the largest groups of structurally related enzymes with diverse catalytic functions. Members in this family include proteases, lipases, esterases, dehalogenases, peroxidases, epoxide hydrolases, and others^11^. These enzymes share a common α/β hydrolase fold and a catalytic triad Ser-His-Asp with the Ser residue acting as the nucleophile. This suggests that they share a common ancestor^11^.

In order to reveal its functional characteristics, we biochemically characterized the enzymatic activity of StnA and studied the structures of apo StnA (SeMet-StnA) and the complex with its substrate (S185A mutant). The structure of StnA has a unique cap domain that contributes nearly all surface area of the binding pocket. This is the first reported case with the specific binding pocket located at the cap domain instead of the interface between the cap domain and the α/β hydrolase fold domain.

## Results

### Enzyme activity of StnA *in vitro*

**Figure 2:**
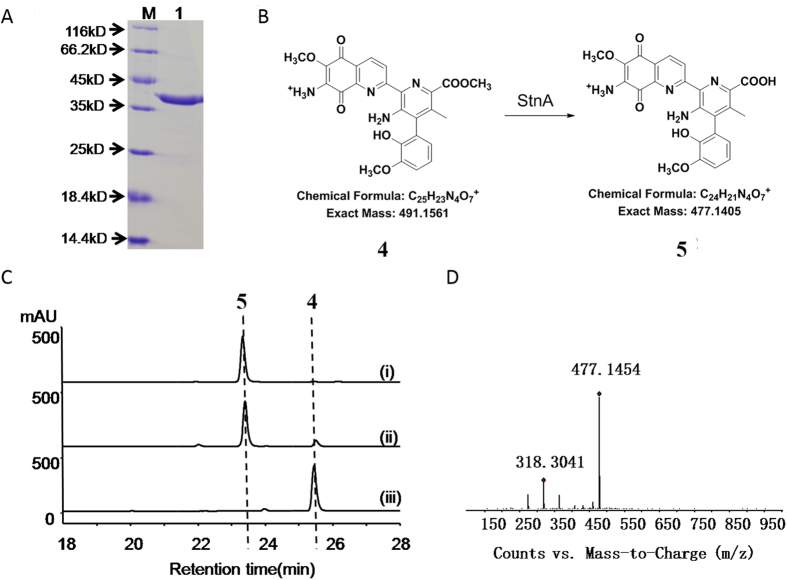
Characterization of StnA *in vitro*. (**A**): SDS-PAGE of recombinant StnA. Lane 1 represents protein marker; lane 2 for purified StnA (42.5 kDa). (**B**): The predicted reaction catalyzed by StnA. (**C**): HPLC analysis of the StnA–catalyzed hydrolysis of **4** at 375 nm. (i) **5** standard, (ii) StnA catalyzed reaction, (iii) negative control with **4** incubated with inactive StnA. (**D**): High-resolution mass analysis of the product of reactions. The reactions were performed in citrate-phosphate buffer pH 6 at 30 °C with 25 nM StnA and 100 μM **4**.

To test the hydrolysis activity of StnA in the production of the compound **5** from the compound **4**, StnA was optimized towards the sequence, subsequently overexpressed in *E. coli* BL21 (DE3) and purified to near homogeneity (Fig. 2A). The sequence analysis reveals that StnA is a member of α/β-fold hydrolase superfamily that does not require any cofactor in general. When **4** was incubated with StnA (25 nM) at room temperature for 30 min, **4** was converted to **5** (Fig. 2B) that was confirmed by comparison with the standard and the analysis of high resolution mass (Fig. 2C & D). At the optimal reaction condition (50 mM citrate-phosphate buffer at pH 6.0 at 30 °C), the steady-state kinetic parameters for StnA with varying concentrations of **4** were determined to be 39.01 ± 7.07 μM for *K*_m_ and 7.86 ± 0.63 s^−1^ for *k*_cat_, respectively.

### Quality assessment of the crystal structures

In order to reveal its functional characteristic, we further studied the crystal structures of StnA. The selenomethionine substituted StnA (SeMet-StnA) was crystallized in the space group C2 with four molecules per asymmetric unit. The structure of the enzyme was determined by single-wavelength anomalous dispersion (SAD) phasing method and refined to 2.71 Å with an R_factor_ of 0.1923 and R_free_ of 0.2196. The final model of StnA consists of residues 61–375 because of the poor electron density for the 60 residues at the N-terminal region. The root mean squared deviations (RMSD) of bond lengths and angles are 0.0166 Å and 1.2498°, respectively, for the refined structure. The average temperature factor (B) is 36.86 Å^2^ (49.11 Å^2^ from the Wilson plot). The stereochemical quality of the model was assessed by Procheck^12^. The ratios of preferred and allowed regions from the Ramachandran plot are 95.71% and 4.05%, respectively^13^. Residue Thr77 in each monomer is in the *cis* conformation. This is in good agreement with the results of electron density (2*Fo-Fc*). The statistics for data collection and refinement is summarized in Table 1.

The S185A complex structure was determined in a different space group P1 with the resolution of 2.90 Å by molecular replacement, using SeMet-StnA as a search model, with 7 molecules in the asymmetric unit. It is possible that there are eight molecules in the asymmetric unit, but it cannot be confirmed due to poor electron density (see Supplementary Fig. S1 and Table S1). The R_factor_ and R_free_ of final model are 0.2437 and 0.2976, respectively. RMSD of bond lengths and angles are 0.0097 Å and 1.3705°, respectively, for the refined structure. The average B factor for all atoms is 51.95 Å^2^ (57.75 Å^2^ from the Wilson plot). The ratios of preferred and allowed regions from the Ramachandran plot are 94.76% and 4.55%, respectively. The overall structures of the free and complex are very similar with a RMSD of 0.232 Å over 316 Cα atoms. Therefore, the structure of molecule A of S185A in complex with substrate STM was used to discuss in following section (see Supplementary Fig. S2).

### Overall structure

**Figure 3:**
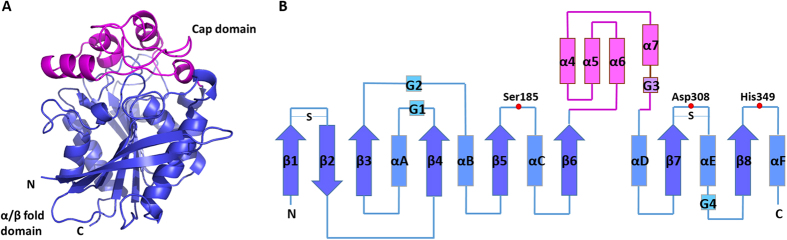
Overall structure of StnA. (**A**): 3D structure of StnA. The α/β-fold domain is colored in slate, while the cap domain in magenta. (**B**): Topology diagram of StnA using the same color scheme as in panel A. The catalytic triad is marked in red node.

The overall three-dimensional structure of monomeric StnA is shown in Fig. 3A. This indicates that StnA structure includes two distinct domains: a nearly globular α/β fold domain (Ser61-Ala208 and Asp289-Gly375) and a cap domain (Leu209-Ile288) that is on the top of the α/β fold domain. Its structural characteristic is similar to those of other esterase. StnA is stabilized by two additional disulfide bonds of Cys61/Cys64 and Cys312/Cys319, which are located between β1 and β2 and between β7 and αD, respectively (Fig. 3B). Both PISA prediction and purification experiment were confirmed that StnA is a monomer in solution.

### Structure of the α/β fold domain

The architecture of the α/β fold domain of StnA structure is similar to those of other hydrolases. Briefly, it consists of a mostly parallel, eight-stranded β-sheet surrounded by six α-helices on both sides with β2 antiparallel to the other strands, which has been regarded as the “canonical” feature of the α/β hydrolase fold family. The eight-stranded β-sheet is highly twisted, and the last strand β8 is oriented with a twisting angle of approximately 90° to β1. There are also three 3_10_-helicies located at β3-αA, β4-αB, and αE-β8. Helices αA, αF, and 3_10_-helix G1 are located on one side of the central β-sheet, and helices αB, αC, αD, αE and the 3_10_-helicies G2, G4 on the other side.

### Structure of the cap domain

**Figure 4:**
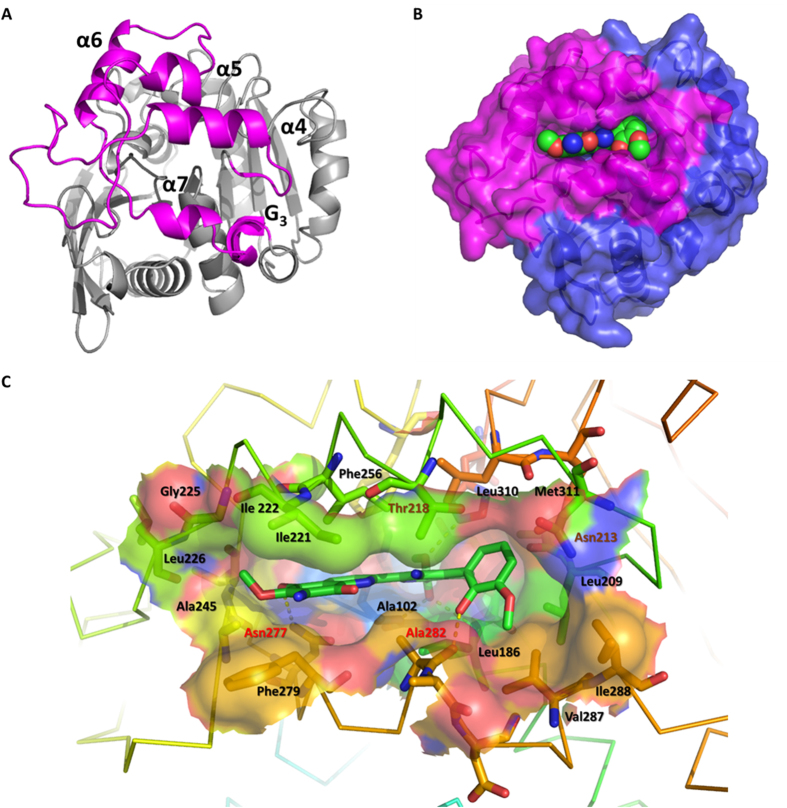
The cap domain and binding pocket of StnA. (**A**): Top view of StnA (the cap domain shown in magenta). (**B**): Binding model of StnA/STM complex. The transparent surface for the α/β-fold domain and the helical cap are differentiated by colors. The substrate STM is displayed as spheres. (**C**): A side view of key residues around the substrate binding pocket in StnA.

The cap domain is constituted by four α-helices (α4, α5, α6 and α7) and one 3_10_-helix G3, which extends from β6 of the α/β fold domain and ends of helix αD (shown in Fig. 4A). Helices α4 and α7 are stacked as antiparallel and participated in the formation of the substrate-binding pocket. The binding pocket is a large flat cavity with a volume of 740.4 Å^3^ and a surface area of 525.9 Å^2^ as estimated by *CASTp*^14^. Most residues in binding pocket are hydrophobic residues, such as Leu209, Ile221, Ile222, Ala245, Phe256, Phe279, Ala282, Val287, Ile288, Leu310, and Met311, except for three polar residues of Asn213, Thr218 and Asn277 (Fig. 4C). Substrate **4** (PDB 3-letter-code is STM) perfectly binds into this narrow pocket. The surface view shows that the binding interaction between STM and StnA is mainly contributed by the cap domain residues (Fig. 4B).

### Active site analysis

**Figure 5:**
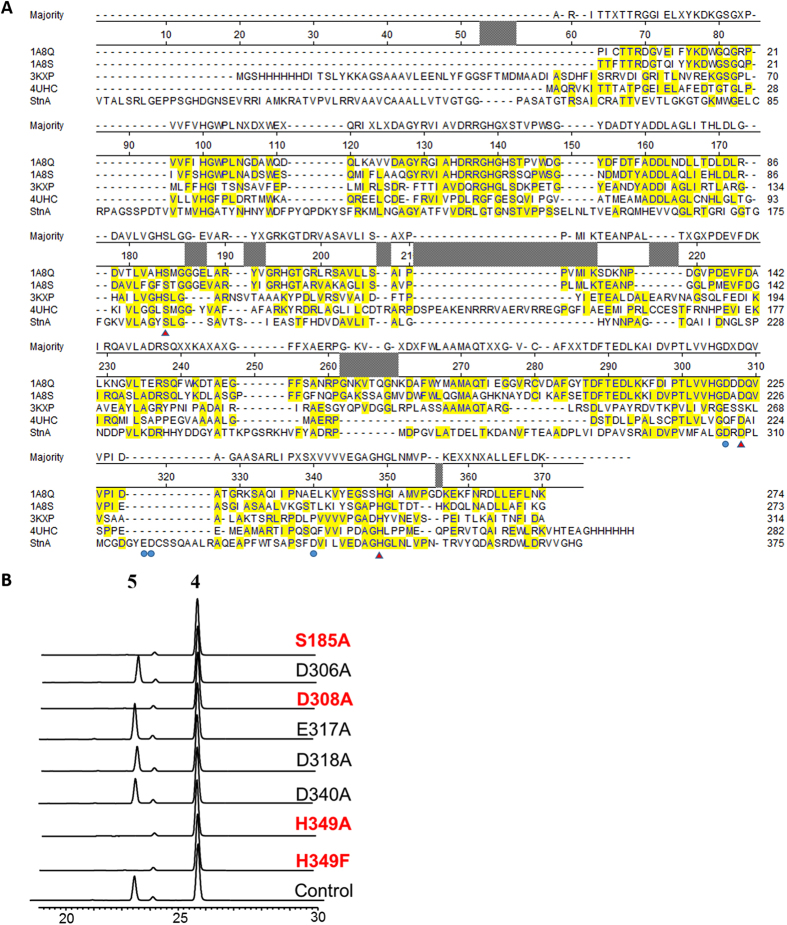
Sequence alignment and HPLC profiles. (**A**): The protein sequence alignment of StnA with bromoperoxidase A1 from *Streptomyces aureofaciens* (PDB ID: 1A8Q)^60^, chloroperoxidase from *Pseudomonas fluorescens* (PDB ID: 1A8S)^60^, succinate hydrolase from *Mesorhizobium loti* (PDB ID: 3KXP)^22^, thermophilic esterase from *Thermogutta terrifontis* (PDB ID: 4UHC)^61^ (the predicted catalytic residues were marked with red triangles and blue nodes). (**B**): HPLC profiles of biochemical assays of the mutants of StnA catalyzed hydrolysis of STM at 375 nm.

The sequence alignment of StnA and its homologous structures suggests that S185, D306, D308, E317, D318, D340, and H349 are likely catalytic residues (Fig. 5A). In order to identify the catalytic triad, a set of site-directed mutants for StnA was constructed and HPLC was used to measure their catalytic activity (Fig. 5B). The data clearly shows that Ser185-Asp308-His349 constitutes the catalytic triad of StnA.

**Figure 6:**
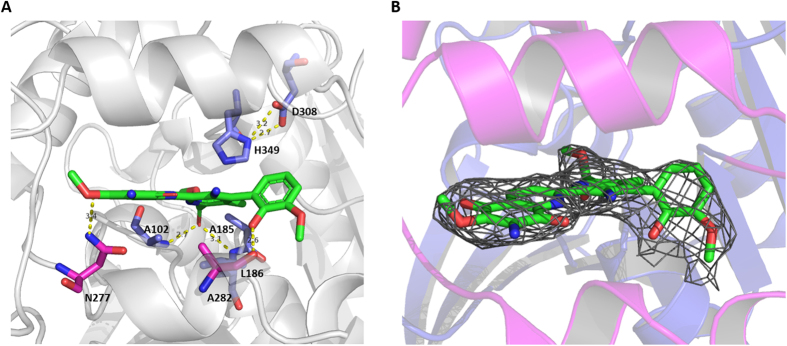
Structure of STM and StnA complex. (**A**): The hydrogen bond network of StnA/STM complex. Substrate and residues Ala102, Ala185, Leu186, Ala282, Asn277, Asp308 and His349 are shown as sticks and hydrogen bond distance between them are shown as yellow dash. (**B**): The *2Fo-Fc* sigma-weighted electron density map (grey mesh) is at 1σ around the substrate STM in the binding pocket.

In the catalytic triad, Ser185 plays the role of nucleophile, His349 is the proton acceptor/donor, and Asp308 is the acidic residue stabilizing the histidine. The positions of Ser185, Asp308, and His349 are similar to those of the canonical fold of the α/β-hydrolase family (Fig. 3B). The catalytic Ser185 is located within a conserved pentapeptide motif, Gly-X-Ser-X-Gly^15^, and is situated at the nucleophile elbow in a sharp turn between β5 and helix αC and positioned at the bottom of the binding pocket. In this unique central location, the serine is effectively shielded from the bulk solvent. The presence of Gly183 and Gly187 is in close proximity to Ser185, preventing steric hindrance and facilitating access to the nucleophile elbow. The catalytic His349, located in the loop between β8 and helix αF, points towards the nucleophile, with its N^ε2^ atom at a distance of 3.2 Å from the O^γ^ atom of Ser185. The His349 N^δ1^ atom forms a hydrogen bond/salt bridge with O^δ1^ (3.2 Å) and O^δ2^ (2.7 Å) of Asp308, located between strand β7 and helix αE (Fig. 6A). The alignment between native and S185A mutant complex was shown in Supplementary Fig. S3. The figure suggests that the catalytic triad is relative stable with small change.

### Substrate-binding cavity of StnA

The electron density for the substrate at the binding pocket is clear in every molecule of the asymmetric unit (Fig. 6B). Ring A, B, and C share a plane and ring D forms a 60° angle to the shared plane. The hydrophobic nature of the binding sites and the aromatic nature of the substrate suggest that the substrate recognition could be predominantly controlled by the shape-complementarity between the substrate and the binding pocket, i.e. hydrophobic interactions.

**Figure 7:**
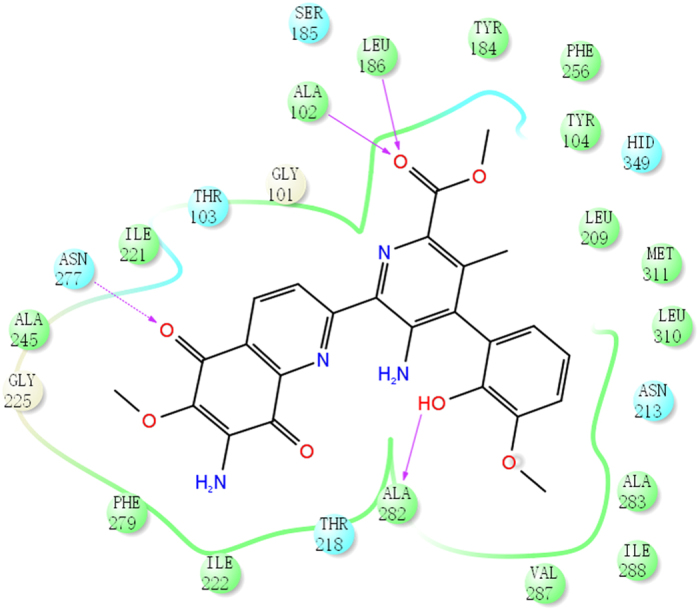
**Representative interactions between substrate STM and the residues on the StnA generated by Maestro 10.6**^62^. The hydrophobic residues were shown in green, the polar residues were in cyans, and the hydrogen bonds were in magenta arrows.

The interactions between STM and StnA were identified by Maestro and shown in Fig. 7, indicating that 16 hydrophobic interactions and four hydrogen bonds exist. Among the four hydrogen bonds, two hydrogen bonds are between the substrate and the main chain amide nitrogen of Ala102 and Leu186 (2.7 Å and 3.1 Å respectively) and play key roles in forming the oxyanion hole (Fig. 6A). Two additional hydrogen bonds are between the main-chain carbonyl group of Ala282 and the hydroxyl group of ring D, and between the side-chain amide group of Asn277 and the carbonyl group of ring A. Furthermore, there is also a possible π-π stacking interaction between ring A and Phe279 (Fig. 4C).

### Molecular dynamics simulation of StnA/STM complex

**Figure 8:**
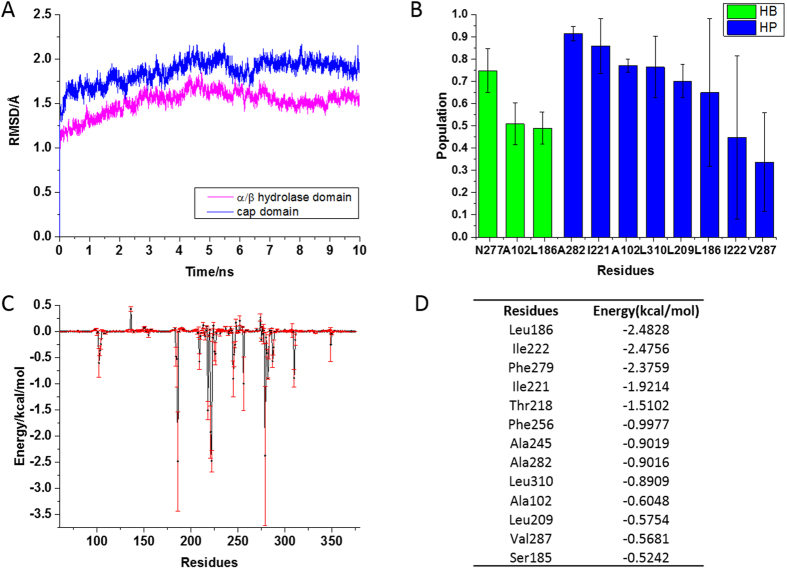
The results of molecular dynamic simulation. (**A**): RMSDs of the α/β hydrolase fold domain and cap domain. (**B**): The hydrogen bond (green) and hydrophobic interaction (blue) between StnA and substrate STM. (**C**): Binding free energy of every residues. (**D**): The binding free energy for the top 13 residues.

Molecular dynamics simulation was further used to gain insight into the binding mode and affinity. The RMSDs relative to initial structure for each domain are shown in Fig. 8A. The results indicate that 10 ns simulations are sufficient for the equilibration at room temperature and the large fluctuations are focused on the cap domain, while the α/β hydrolase domain is relatively stable. The superposition between the initial structure and last snapshot structure was shown in Supplementary Fig. S4. This indicates that STM can stable locate at the binding pocket of StnA.

In order to reveal the driving forces in substrate binding, the interactions between STM and StnA are shown in Fig. 8B. Three hydrogen bonds were found with population higher than 40%, mainly focused on Asn277, Ala102, and Leu186. Seven stable hydrophobic contacts were found between STM and Ala282, Ile221, Ala102, Leu310, Leu209, Leu186, Ile222, with population higher than 40%, which may enhance the binding affinity between STM and StnA. The binding free energy between STM and StnA was further calculated to understand the binding interaction in more details. The averaged binding free energy of StnA/STM was estimated to be −41.10 ± 3.17 kcal/mol by the MMPBSSA method. Residue decomposition of the binding free energy was also conducted and shown in Fig. 8C,D. It was found that Leu209, Thr218, Ile221, Ile222, Ala245, Phe256, Asn277, Phe279, Ala282, Val287 are located in the cap domain, suggesting a key role of the cap domain in stabilizing the substrate binding. The dominance of hydroponic residues on the top of contributor list also indicates the stabilizing interaction by the cap domain is hydrophobic in nature.

### Binding assay of StnA and mutants

**Figure 9:**
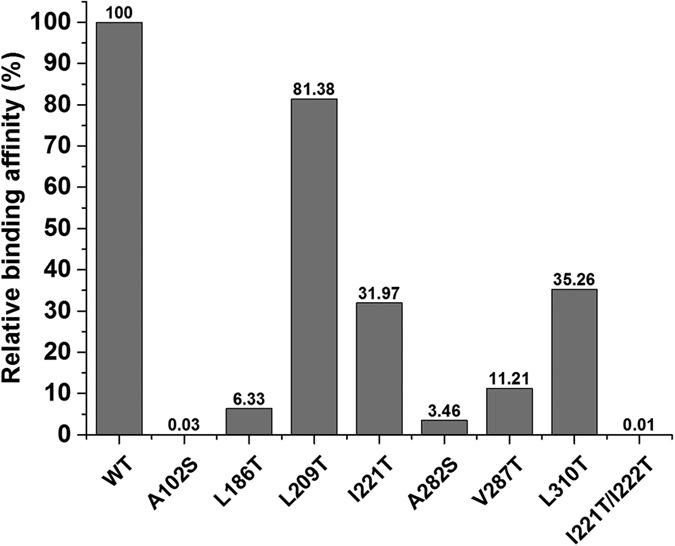
Relative binding affinity of WT and mutants. The wild type of StnA exhibited 100% binding affinity, and the mutants showed the relative percent of binding affinity.

In order to confirm the binding mode between StnA and substrate STM, the hydrophobic residues which have interacted with STM were mutated to hydrophilic residues and the binding assay of these mutants was performed by Octet RED biolayer interferometry system. The binding affinity of wild type (WT) and mutants was shown in Fig. 9 and Supplementary Table S2. In contrast to WT of StnA, which exhibited 100% binding affinity, the A102S, L186T, A282S, V287T, and I221T/I222T mutants dramatically decreased the binding activity. Moreover, I221T and L310T point mutants resulted in a 3-fold decrease in binding affinity for STM. L209T point mutant also shows slightly lower binding affinity than WT. These results are consistent with those of molecular dynamics simulation that these hydrophobic residues play key roles in binding affinity (Fig. 8B).

## Discussion

Lipolytic enzymes such as esterases (EC 3.1.1.1) and lipases (EC 3.1.1.3) represent a group of hydrolases catalyzing both hydrolysis and synthesis of ester bonds and are widely distributed in animals, plants, and microorganisms. The lipolytic enzymes belong to serine hydrolysis superfamily. Therefore, the activity of these enzymes mainly depends on a highly conserved catalytic triad of Ser-His-Asp^16^. Because of their broad substrate specificity, stability in extreme environment, and stereoselectivity, the enzymes are widely used as biocatalysts^17^,^18^. Therefore, identification of novel esterases and lipases will increase the diversity of lipolytic enzymes and help in selection of suitable biocatalysts for challenging reactions^19^.

The α/β hydrolase fold domain provides a stable scaffold for the active sites of lipolytic enzymes. The main difference of these enzymes is focused on the cap domain^11^, which plays an essential role in the substrate specificity. In general, the substrate is bound at an internal cavity and/or large surface cleft between the interfaces of these two domains^20^.

In order to compare binding modes among different hydrolases, structural homologues of StnA were aligned with DALI^21^ and are shown in Table 2. The sequence identity between these hits and StnA is equal to or less than 20% and RMSD is at least 2.4 Å. Note that if only the α/β hydrolase core domain was used in the search, many more close homologues can be found with RMSD’s as low as 1.5 Å (data not shown). In contrast, we could not find any hits for the isolated cap domain. These results suggest that the cap domain in StnA may be very unique.

**Figure 10:**
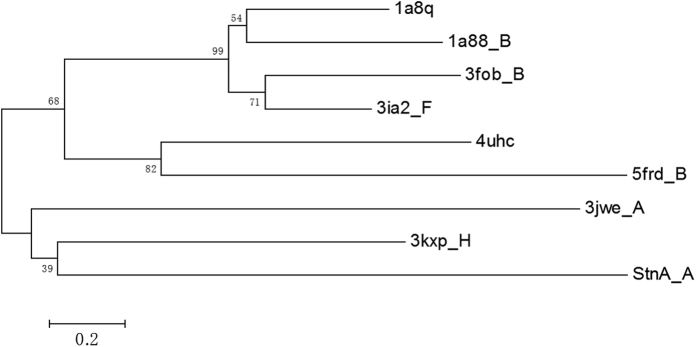
Phylogenetic tree of StnA and other homologous enzymes. The tree was constructed by VMD^34^ and MEGA^63^.

**Figure 11:**
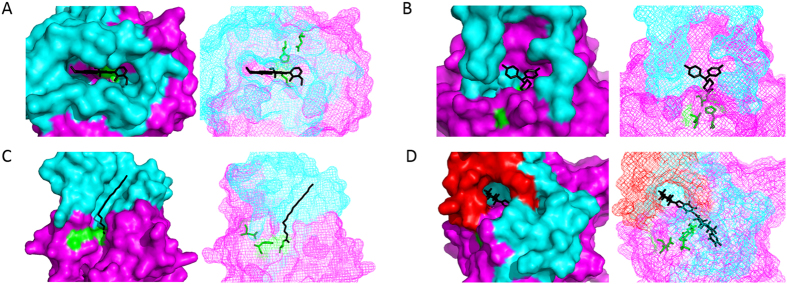
The surface (left) and mesh (right) schematic diagram of binding pocket. (**A**). StnA; (**B**). 3JWE; (**C**). 1EH5; (**D**). 3PE6. The cap domain, α/β hydrolase domain, catalytic triad, and movable lid are shown as cyan, magenta, green, and red, respectively. Ligands are shown in black sticks.

In order to reveal the evolution of StnA, the phylogenetic tree of StnA and these homologues structures was constructed and shown in Fig. 10. The results show that StnA is clustered in a distinct clade and is closest to succinate hydrolase from *Mesorhizobium loti* (3KXP)^22^ and human mono-glyceride lipase (3JWE)^23^. The binding pockets of both 3KXP and 3JWE are located between the interfaces of α/β hydrolase domain and cap domain. 3JWE is a membrane-interacting protein and its inhibitor binding site is located between the two domains. However the binding pocket in StnA is mainly composed of the cap domain, with only the catalytic triad and oxyanion hole are from the α/β fold domain (Fig. 11A,B).

As a broad type of α/β-fold hydrolases, lipolytic enzymes can hydrolyze water-insoluble ester substrates at binding pockets near the water/lipid interface. The active sites of most lipolytic enzymes are buried under secondary structure elements, including a narrow tunnel, or a flap as a flexible lid for the entrance of the substrate and the release of the product^16^. An eukaryotic thioesterase (PDB ID: 1EH5, Fig. 11C)^24^ has a bend tunnel formed by two domains as entry and exit routes for the substrate and product. Another human lipase (PDB ID: 3PE6, Fig. 11D)^25^ has a movable lid, formed by α4 and part of the loop connecting to α5, which acts as a highly dynamic open and close conformations during ligand binding and release. The cap domain may participate in the process of product release. However, StnA is significantly different from the lipolytic enzymes without open and close conformations or long tunnel. Based on the analysis of sequence, structure, and phylogenetic tree, we propose that StnA be classified as a new subfamily of lipolytic enzymes.

## Conclusion

StnA is an essential enzyme in the Streptonigrin biosynthesis pathway. The crystal structure shows that StnA has an α-helical cap domain positioned atop a common α/β hydrolase domain. The substrate STM binds close to the catalytic triad (Ser185-His349-Asp308) in a hydrophobic pocket which mainly composed by cap domain. Hydrolysis mechanism is the same as other esterase with Ser185 acting as nucleophile and transition state stabilized by an oxyanion hole formed by the backbone amides of residues Ala102 and Leu186. The binding specificity appears to be controlled mainly by the shape complementarity between the substrate and the binding pocket. The binding stability is dominated by hydrophobic interactions and confirmed by molecular dynamics simulation and mutagenesis experiment. Our structural data shows that a new subfamily lipolytic enzyme can be proposed due to its specific substrate-binding mode.

## Methods and Material

### Cloning, expression and purification of StnA

The *stnA* gene was amplified using the following primers: *stnA*-exp-F 5′-CCTTAGGATCCATGGAACGTGCTACC-3′ (underlined *BamH*I restriction site) and *stnA*-exp-R 3′-CCATCTCGAGACCGTGACCAACAACACG-5′ (underlined *Xho*I restriction site). The PCR product was ligated into the pBluescript II SK (+) (Stratagene) vector and digested with *EcoR*V. The recombinant plasmid was digested with *BamH*I and *Xho*I to generate a 1.1 kb fragment, and inserted into the corresponding site of pET28a (Novagen) vector with both N- and C-terminal His-tag. The StnA point mutants were constructed by the whole-plasmid PCR and *Dpn*I digestion method, and were verified by plasmid sequencing.

WT StnA and various point mutants proteins were overexpression in *Escherichia coli* BL21 (DE3) cells (Novagen). Cell were grown at 37 °C in LBBS medium (10 g/L Tryptone, 5 g/L Yeast Extract, 5 g/L NaCl, 182 g/L Sorbitol, and 0.3 g/L Betaine) supplemented with 50 μg/mL kanamycin until an OD_600_ of 0.8–1.0, and were then induced for 20 h at 16 °C with 0.4 mM IPTG. Cells were harvested by centrifugation at 4 °C, and the cell pellet was resuspended in ice-cold binding buffer (25 mM Tris-HCl, pH 8.0, 300 mM NaCl, and 20 mM imidazole). Resuspended cells were lysed by sonication, followed by centrifugation. The resulting supernatant of cell lysates was than purified on Ni^2+^-NTA affinity chromatography (Qiagen). Then the protein was further purified by SOURCE 15Q ion exchange column (GE Healthcare) and Superdex 200 gel filtration chromatography (GE Healthcare). The final buffer contained 10 mM Tris-HCl, pH 8.0, 100 mM NaCl, 2 mM DTT and 1 mM EDTA. Peak fractions were combined with a final concentration of 10 mg/ml, flash-frozen in liquid nitrogen, and stored in −80 °C until use. The protein purity was assessed by SDS-PAGE analysis and concentration was determined using the Bradford assay with Bovine serum albumin as a standard.

Selenomethionine substituted StnA protein (SeMet-StnA) was expressed using the methionine autotrophic *E. coli* B834 (DE3) cultured in the M9 medium and purified as before, except that all buffers contained 20 mM dithiothreitol (DTT).

### Biochemical assay of StnA

All the reactions were performed in citrate-phosphate buffer pH 6 at 30 °C with 25 nM StnA and 100 μM **4**. These experiments were performed in triplicates. The products were analyzed with High Performance Liquid Chromatography (HPLC). The HPLC analysis was conducted using HPLC system (Agilent 1200 series, Agilent technologies) coupled with a ZORBAX SB-C18 (Agilent, 5 μm, 4.6 × 150 mm) column at the flow rate of 0.3 mL/min with detection at 210 nm, 245 nm, and 375 nm. For the analysis of the StnA-catalyzed hydrolytic reactions of **4**, HPLC analysis was performed using a 25 min gradient from 10–100% CH_3_CN containing 0.1% formic acid in H_2_O containing 0.1% formic acid. High-resolution mass was performed using Agilent 6530 Accurate-Mass Q-TOF LC-MS spectrometer coupled with an Agilent HPLC 1200 series. The kinetic parameters of StnA-catalyzed hydrolysis of **4** were determined in the condition including 20 nM StnA, 50 mM citric acid/Na_2_HPO_4_ buffer (pH 6.0), and **4** with varying concentrations from 11 to 85 μM in a 100 μl final volume. The data were analyzed using the Michaelis–Menten equation, and the reported error indicated the standard deviation among the three replicates.

### Crystallization and data collection

Protein crystals were grown by hanging-drop vapor diffusion method at 14 °C. 1 μl of SeMet-stnA solution was mixed with 1 μl of reservoir solution containing 0.1 M HEPES, pH 7.5 and 60% MPD (2-methyl-2,4-pentanediol) (Hampton Research Co.) and equilibrated against 200 μl of reservoir solution. Crystals of 0.05 mm × 0.05 mm × 0.2 mm were grown in two weeks. StnA S185A/ATM complex crystal was obtained in 0.1 M ammonium Citrate dibase, 0.1 M sodium Citrate, pH 5.0, 20% 2-propanol, 15% PEG 3350, and 4% ethylene glycol (Hampton Research Co.). Substrate STM was added in a molar ratio of 1:2 (protein:substrate). For cryoprotection, the crystals were transferred to crystallization solution supplemented with 20% (v/v) glycerol. The crystals were mounted in a cryoloop and subsequently flash-cooled in liquid nitrogen.

X-ray diffraction data sets of SeMet-StnA and StnA S185A/STM complex were collected at 100 K under a nitrogen stream on an ADSC Quantum 315 R CCD area detector at the BL17U1 beamline at Shanghai Synchrotron Radiation Facility (SSRF). Datas were further processed and scaled using HKL2000 software^26^ (Table 1).

### Structure determination and refinement

SeMet-StnA crystal was found to belong to space group C2 and contained four molecules in the asymmetric unit, determined at 2.7 Å resolution. The single-wavelength anomalous dispersion (SAD) phases were determined by using the Autosol module of PHENIX^27^. After the model-building by Coot^28^ and refinement by CCP4 program REFMAC^29^,^30^,^31^, the final model had an R/R_free_ value of 19.23%/21.96% and included StnA residues 61–375. In the Ramachandran plot^13^, there are 95.71%, 4.05% and 0.24% of residues in the preferred, allowed and outliers regions respectively.

StnA S185A/STM complex crystal belongs to the space group P1, with seven molecules in the asymmetric unit. Its structure was determined to 2.9 Å by molecular replacement method with CCP4 program PHASER^29^,^32^, using the structure of SeMet-StnA structure as the searching model. The final model had an R/R_free_ value of 24.37%/29.76%. In the Ramachandran plot, there are 94.76%, 4.55% and 0.68% of residues in the preferred, allowed and outliers regions respectively. Model qualities were validated by CCP4 program PROCHECK^12^, while calculations of binding pocket surface and volume were performed with *CASTp*^14^. All the figures were prepared using the program PyMOL^33^.

Coordinates and structure factors have been deposited in the RCSB Protein Data Bank (PDB) with accession codes 5HDF for SeMet-StnA and 5HDP for StnA S185A/STM complex.

### Phylogenetic analysis

Amino acid sequence and structures were obtained from PDB database and aligned with VMD program^34^. Phylogenetic tree was inferred by the Neighbor-Joining method, as implemented in the MEGA 6 program package^35^. The order of branching was generally supported by bootstrap method based on 500 replication. The evolutionary distances were computed using Jones-Taylor-Thornton (JTT) matrix-based method and are in the units of the number of amino acid substitutions per site. All positions containing gaps and missing data were eliminated.

### Molecular dynamic simulation

Three-dimensional structures of substrate STM was built and optimized with SYBYL modeling program (SYBYL-X v1.0 Tripos). The Tripos force field^36^ was applied to perform energy minimization for these structures.

The Amber 12 package^37^ and the *ff99IDPs* force field^38^,^39^,^40^ were used to perform MD simulations. Initial coordinates of wide type was corresponding to S185A/STM complex crystal structure using PyMOL. Antechamber module^41^ was applied to handle the force field of the ligands and AM1-bcc charges were assigned to the ligands. Counter-ions were added to maintain system neutrality. SHAKE algorithm^42^ was implemented to constrain the bonds involving hydrogen atoms. All systems were solvated in a truncated octahedron box of the TIP3P water model^43^ with a buffer of 10 Å. The Partial Mesh Ewald (PME) method^44^ was used to evaluate long range electrostatic interaction. 1000-step steepest descent minimization was performed to relieve any structural clash in the solvated systems. This was followed by heating up and brief equilibration for 20 ps in the NVT ensemble at 298 K with PMEMD of AMBER12. Langevin dynamics with a time step of 2 fs were used in the heating and equilibration runs with a friction constant of 1 ps^−1^. To collect enough snapshots for statistically meaningful structural analysis, up to three independent trajectories of 10.0 ns were collected to analyse their structural properties.

CPPTRAJ^45^,^46^ was used to process the trajectories. Hydrophobic interaction and hydrogen bond assignment were handled with the in-house perl script^39^,^47^,^48^,^49^. Hydrophobic interaction is defined as the distance between the center mass of side chain for the hydrophobic residue and the ring center of A, B, C, and D for the ligand less than 6.5 Å. The hydrogen bond is defined when the distance between donor and acceptor atoms less than 3.5 Å and the hydrogen bonding angle larger than 120°. Binding free energies were calculated with the MM/PBSA method (python script) from the Amber 12 package for the equilibrium conformers^50^,^51^,^52^,^53^,^54^,^55^,^56^,^57^,^58^,^59^. All figures were plotted using OriginPro 9.1.

### Binding assay of wild type and mutants for StnA

The binding assay was carried out using Ni-NTA biosensors on an Octet RED biolayer interferometry system (FortéBio) that measures changes in layer thickness (in nm) in real time. All the steps of the assay process were performed at 30 °C with the plate shaking speed set at 1000 rpm. Solid-black 96-well microplates (Greiner Bio) were made up using 200 μl volumes. Wild type and mutants prepared at 0.3 mg/ml in running buffer (25 mM HEPES, pH 7.5, 250 mM NaCl, 40% (v/v) DMSO) and substrate STM was dissolved in DMSO at 1 mg/ml. Firstly, a set of sensors were rinsed in running buffer for 180s which served as the baseline. Secondly, sensors were immobilized for 300 s with each protein solution. Thirdly, sensors were washed in running buffer for other 180 s. Then, sensors were exposed to substrate STM solution for 300s in association step. Finally, the dissociation step was carried out a 300s time period in running buffer.

Binding kinetics were calculated using the Data Analysis v7.1 software (FortéBio). The running buffer blank was used as a reference cell subtraction and the association (*k*_a_) and dissociation (*k*_d_) rate constants were obtained by fitting the 1:1 binding interaction model. Binding affinity constant *K*_D_ was calculated by the ratio of the *k*_d_/*k*_a_.

## Additional Information

**How to cite this article**: Qian, T. *et al*. Crystal Structure of StnA for the Biosynthesis of Antitumor Drug Streptonigrin Reveals a Unique Substrate Binding Mode. *Sci. Rep.* **7**, 40254; doi: 10.1038/srep40254 (2017).

**Publisher's note:** Springer Nature remains neutral with regard to jurisdictional claims in published maps and institutional affiliations.

## Supplementary Material

Supplementary Information

## Figures and Tables

**Figure 1:**
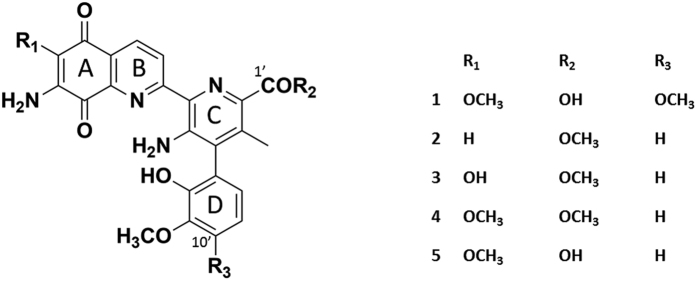
Chemical structures of streptonigrin (STN) and related compounds.

**Table 1:** Data collection and refinement statistics.

Structures	SeMet-StnA	Complex
**Data collection**		
Wavelength (eV/Å)	12660.8/0.9819	12662/0.9818
Resolution (Å)	2.7	2.9
Space group	C 2	P 1
Cell-unit parameters	a = 178.42 Å,b = 81.97 Åc = 118.69 Å	a = 83.26 Åb = 92.85 Åc = 104.04 Å
	α = 90.00°β = 126.75°γ = 90.00°	α = 115.06°β = 106.09°γ = 97.69°
Matthew’s coefficient	2.57	2.68
% solvent	51.86	54.21
No. mol. per ASU	4	7
No. observations	140343	175574
No. unique reflections	37407	54912
Redundancy	3.8	3.2
R_merge_ (%)	0.111 (0.300)	0.153 (0.487)
Mean I/σ	14.55 (6.08)	6.12 (2.54)
Completeness (%)	99.7	95.4
**Refinement**		
R_working_ (%)	19.23	24.37
R_free_ (%)	21.96	29.76
Figure of merit	0.8592	0.7588
No. of atoms	9647	17000
Protein	9595	16720
Ligands		252
Waters	52	28
B-factor (Å^2^)	36.95	51.95
Wilson plot	49.11	57.75
RMSD		
Bond lengths (Å)	0.0166	0.0097
Bond angles (°)	1.2498	1.3705
Ramachandran plot		
Preferred (%)	1205 (95.71%)	2081 (94.76%)
Allowed (%)	51 (4.05%)	100 (4.55%)
Outliers (%)	3 (0.24%)	15 (0.68%)
**PDB ID**	5HDF	5HDP

**Table 2:** Structural homology of StnA to other enzymes, sorted by Z-score.

No.	PDB ID	Z-score*	RMSD*	% id*	Nres*	Description
1	4d9j-C	26.1	3.0	20	438	Designed protein
2	1a88-B	26.1	2.5	17	275	Chloroperoxidase
3	3ia2-F	26.1	2.5	18	271	Arylesterase
4	1a8q-A	26.1	2.5	14	274	Bromoperoxidase
5	4uhc-A	25.9	2.5	19	278	Esterase
6	1k8q-A	25.8	2.7	11	377	Lipase
7	3fob-B	25.7	2.5	18	276	Bromoperoxidase
8	4 × 00-A	25.5	2.7	17	273	Putative Hydrolase
9	1hlg-A	25.4	2.6	14	368	Lipase
10	3kxp-H	25.3	2.5	17	268	Hydrolase
11	2xua-A	25.2	2.8	13	260	Lactonase
12	1hl7-B	25.1	2.8	17	279	Lactamase
13	4dgq-C	25.0	2.6	16	277	Chloroperoxidase
14	3jwe-A	25.0	2.4	15	271	Lipase
15	4f0j-A	24.8	2.7	15	312	Hydrolytic Enzyme
16	4l0c-A	24.5	2.6	14	255	Deformylase
17	3hys-A	24.1	2.8	13	275	Putative Bromoperoxidase
18	5frd-B	24.0	2.5	15	252	Caeboxylesterase
19	1u2e-A	23.8	2.8	16	286	Hydrolase
20	3kda-D	23.6	3.1	14	298	CFTR Inhibitory Factor

^*^Z-score: statistical significance of a match in terms of Gaussian statistics.

^*^RMSD: root mean square deviations

^*^% id: identity

*Nres: superposed Cα atoms.
