# Supplementary material for: Crystal Structure of StnA for the Biosynthesis of Antitumor Drug Streptonigrin Reveals a Unique Substrate Binding Mode
Source: Sci Rep. 2017 Jan 11;7:40254. doi: 10.1038/srep40254 (PMC5225493; doi:10.1038/srep40254)
Supplement: Supplementary Information [file srep40254-s1.pdf]

Supplementary Information

Crystal Structure of StnA for the Biosynthesis of Antitumor Drug Streptonigrin Reveals a Unique Substrate Binding Mode

Tianle Qian,[1] Jing Wo,[1] Yan Zhang, [1,2] Quanwei Song[1,3], Guoqiang Feng[3], Ray Luo,[4] Shuangjin Lin,[1,*] Geng Wu,[1,*] and Hai-Feng Chen[1,5,*]

[1]State Key Laboratory of Microbial metabolism, School of Life Sciences and Biotechnology, Shanghai Jiao Tong University, 800 Dongchuan Road, Shanghai, 200240, China

[2]Shanghai Institute of Immunology, Shanghai Jiao Tong University School of Medicine, 280 South Chongqing Road, Shanghai, 200025, China

[3]Key laboratory of Pesticide and Chemical Biology of Ministry of Education, College of Chemistry, Central China Normal University, 152 Luoyu Road, Wuhan 430079, China

[4]Departments of Molecular Biology and Biochemistry, Chemical Engineering and Materials Science, and Biomedical Engineering, University of California, Irvine, California 92697-3900, USA

[5]Shanghai Center for Bioinformation Technology, 1278 Keyuan Road, Shanghai, 200235, China

*****Corresponding authors
Email addresses: linsj@sjtu.edu.cn; geng.wu@sjtu.edu.cn; haifengchen@sjtu.edu.cn
Tel: 86-21-34204348
Fax: 86-21-34204348.

The authors declare that there is no conflict of interest.

Supplementary Figures and Tables

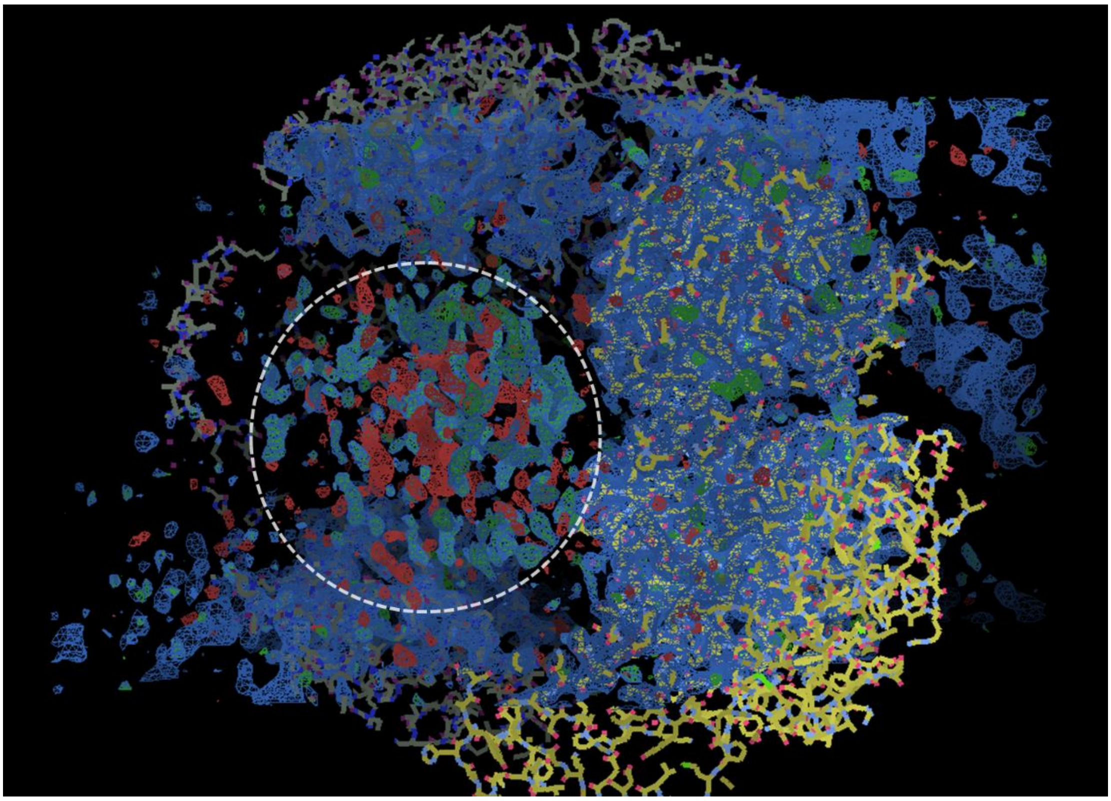

Figure S1. Crystal packing along with the density of S185A complex. The likely position of the eighth molecule in the asymmetric unit is shown in white dash circle.

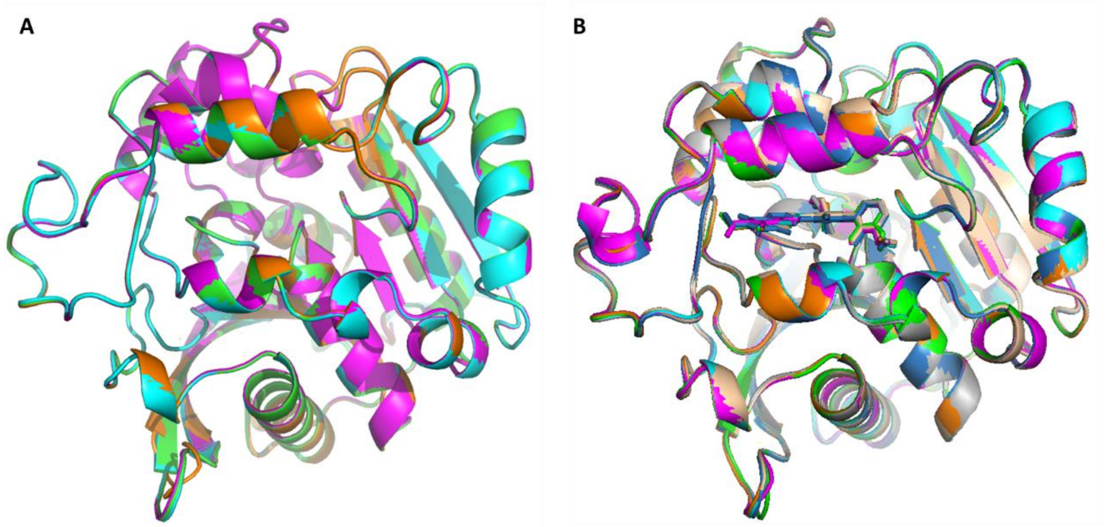

Figure S2. The superposition of all molecules in the asymmetric unit. A: Align four molecules' structures of SeMet-StnA. B: Align seven molecules' structures of S185A complex. The structures were refined with NCS restraints and the RMSDs of each molecule are smaller than 0.076 Å and 0.165 Å for free and complex, respectively. Therefore, we choose the molecule B of SeMet-StnA and the molecule A of complex with substrate STM in text.

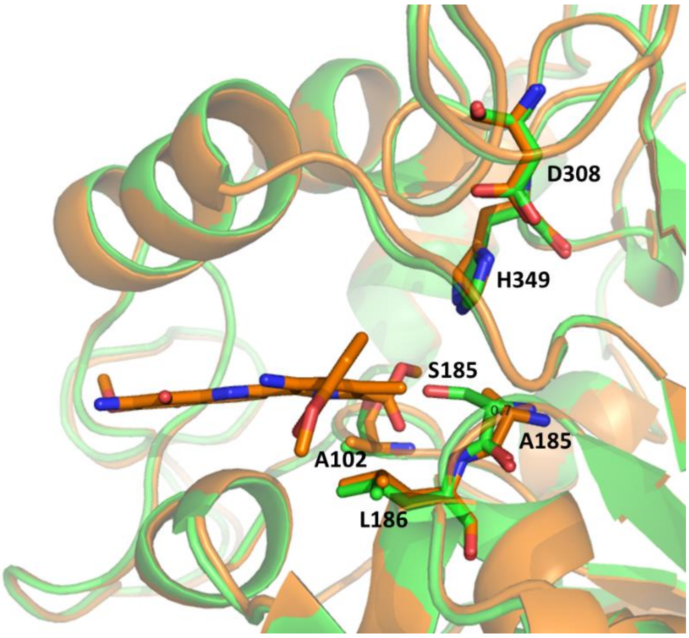

Figure S3. Close-up view of StnA active sites between free (green) and S185A complex (orange) structures. The change of the catalytic triad is small.

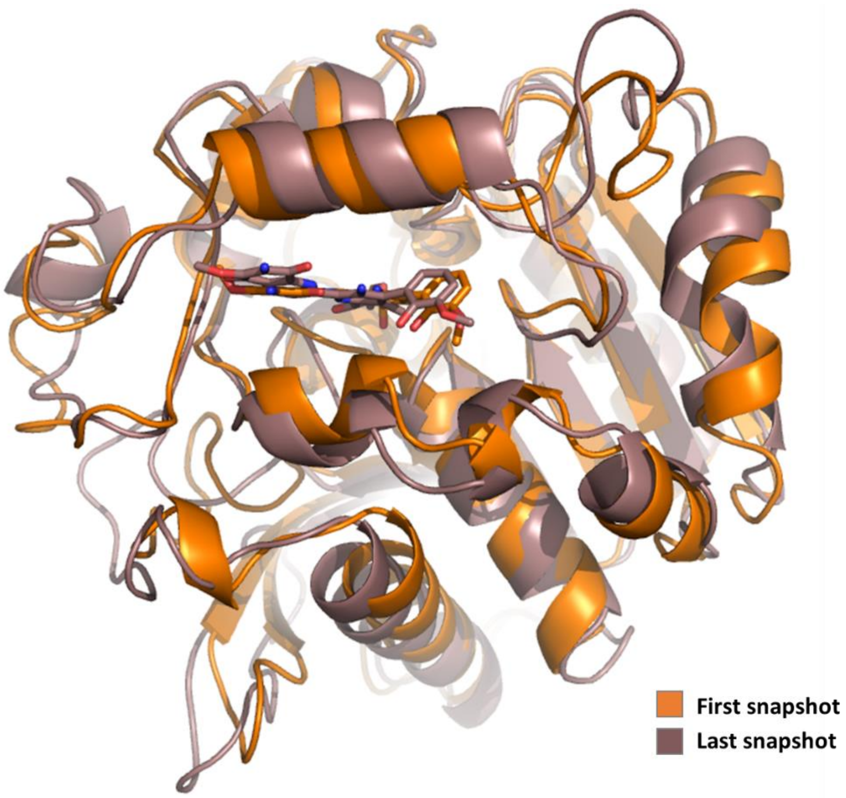

Figure S4. Comparison of StnA complex structures between the first snapshot (orange) and the last snapshot (dark salmon) of molecular dynamics simulation.

Table S1. Solvent content of complex crystal performed with CCP4 program

No. of molecules/asymmetric	Matthew's coefficient	% solvent
**7**	2.68	54.21
**8**	2.35	47.67

Table S2. Kinetic parameters of binding assay

**Protein**	k_a (M^{-1}•s^{-1})	k_d (s^{-1})	K_D (M)
**WT**	$(5.27\pm1.75)\times10^{2}$	$(7.02\pm0.31)\times10^{-3}$	1.33×10^{-5}
**A102S**	$(5.63\pm0.61)\times10^{1}$	2.58 ± 1.06	4.57×10^{-2}
**L186T**	$(3.24\pm1.61)\times10^{1}$	$(6.83\pm15.0)\times10^{-3}$	2.11×10^{-4}
**L209T**	$(9.42\pm0.76)\times10^{1}$	$(1.54\pm0.06)\times10^{-3}$	1.64×10^{-5}
**I221T**	$(7.89\pm0.96)\times10^{1}$	$(3.29\pm0.13)\times10^{-3}$	4.17×10^{-5}
**A282S**	$(6.95\pm0.54)\times10^{1}$	$(2.68\pm0.70)\times10^{-2}$	3.85×10^{-4}
**V287T**	$(1.26\pm0.36)\times10^{2}$	$(1.50\pm0.07)\times10^{-2}$	1.19×10^{-4}
**L310T**	$(3.12\pm1.61)\times10^{2}$	$(1.18\pm0.05)\times10^{-2}$	3.78×10^{-5}
**I221T/I222T**	$(2.83\pm1.24)\times10^{1}$	3.83 ± 1.26	1.36×10^{-1}
